# Incidence and risk factors for metabolic syndrome among urban, adult Sri Lankans: a prospective, 7-year community cohort, follow-up study

**DOI:** 10.1186/s13098-019-0461-7

**Published:** 2019-08-14

**Authors:** Shamila T. De Silva, Madunil A. Niriella, Dileepa S. Ediriweera, Dulani Kottahachchi, Anuradhani Kasturiratne, Arjuna P. de Silva, Anuradha S. Dassanayaka, Arunasalam Pathmeswaran, Rajitha Wickramasinghe, N. Kato, H. Janaka de Silva

**Affiliations:** 1 Department of Medicine, Faculty of Medicine, University of Kelaniya, Thalagolla Road, Ragama, 11010 Sri Lanka; 2 University Medical Unit, Colombo North Teaching Hospital, Ragama, Sri Lanka; 3 National Center for Global Health and Medicine, Toyama, Shinjuku-ku, Tokyo, Japan

**Keywords:** Incidence, Metabolic syndrome, Obesity, Diabetes, Hypertension, Dyslipidemia, Risk factors, Sri Lanka

## Abstract

**Background:**

The metabolic syndrome (MetS) is a clustering of abdominal obesity, diabetes and prediabetes, high cholesterol and high blood pressure, that confers an increased risk of cardiovascular disease. There is limited data on incidence of MetS from South Asia. This study investigated incidence and risk factors for new onset MetS in an urban adult Sri Lankan population.

**Methods:**

Subjects (selected by age-stratified random sampling from the Ragama Medical Officer of Health area) were screened initially in 2007 (35–64 years) and re-evaluated in 2014 (42–71 years). On both occasions they were assessed by structured interview, anthropometric measurements, liver ultrasound, and biochemical/serological tests. MetS was diagnosed on International Diabetes Federation (IDF-2006) criteria. Total body fat (TBF) and visceral fat percentage (VFP) were measured in 2014, using body impedance method. Incidence and factors at baseline, associated with new onset MetS, were investigated among those who presented for re-evaluation.

**Results:**

2985 (99.1%) [1636 (54.8%) women (54.8%); median age (IQR) 53 (47–59) years] from the initial cohort in 2007 had complete data. 2148 (71.9%) [1237 (57.6%) women; median age (IQR) 60 (54–66) years] attended follow-up. 949 of them [701 (73.9%) women; median age (IQR) 60 (54–65) years] had MetS (prevalence 47.2%, 95% CI 45.0–49.4%). Of 1246 who did not have MetS in 2007, 265 [178 (67.1%) women, median age (IQR) 57 (51–64) years] had developed MetS after 7 years (annual incidence 3.5% (95% CI 2.4–4.5%). Females (OR = 4.9, 95% CI 3.4–7.4), BMI > 23 kg/m^2^ in 2007 (OR = 1.6 per unit increase, 95% CI 1.5–1.7), weight gain (by 2–5% OR = 2.0, 95% CI 1.1–3.5; by > 5% OR = 2.2, 95% CI 1.4–3.4), and increase in waist circumference (by 2–5% OR = 7.0, 95% CI 4.0–12.2; by > 5% OR = 13.4, 95% CI 8.3–22.4) from baseline and presence of non-alcoholic fatty liver disease (NAFLD) in 2007 (OR = 1.70, 95% CI 1.04–2.76) were associated new onset MetS. Those with MetS had abnormal VFP and TBF in 2014 [P < 0.001].

**Conclusion:**

In this study, annual incidence of MetS was 3.5%. Female gender, BMI > 23 kg/m^2^ and NAFLD in 2007 and increase in weight and waist circumference from baseline were significantly associated with new onset MetS. Obesity was the best predictor of future MetS.

## Background

The metabolic syndrome (MetS) is a clustering of abdominal obesity, diabetes and prediabetes, high cholesterol and high blood pressure [1]. Since each component of the MetS is an independent risk factor for cardiovascular disease (CVD), when these components occur together the risk of CVD also increases exponentially. Individuals with MetS are twice as likely to die from cardiovascular or cerebrovascular disease, three times as likely to have a myocardial infarction or stroke, and five times more likely to develop type 2 diabetes, compared to those without MetS [2]. Therefore, there are medical and economic imperatives for early identification of individuals likely to develop MetS in later life, since lifestyle interventions and treatment may prevent the development of future diabetes or CVD.

According to estimates of the International Diabetes Federation (IDF) almost one quarter of the world’s adults have MetS [1]. The prevalence of MetS is increasing in most countries, in association with the twin global epidemics of obesity and diabetes. While worldwide prevalence is estimated at 20–25% [3], in the Asia–Pacific region it is 12–37% [4], and in South Asia it is approximately 26% [5]. Previous studies have estimated a MetS prevalence of 24.3% among adults in Sri Lanka [6].

While there are many reports on the prevalence of MetS, data on incidence are scarce. Only a few community based studies have reported on incident MetS in Asia; incidence in West Asia is estimated at 5.5% [7], and in South Korea it is 4.9% [8]. Despite high prevalence of the condition, there are no reports on the incidence or risk factors associated with new onset MetS in South Asian populations.

The Ragama Health Study (RHS) is a large community-based cohort study on non-communicable diseases [9]. It is a collaborative study between the National Centre for Global Health and Medicine, Tokyo, Japan and the Faculty of Medicine, University of Kelaniya, Ragama, Sri Lanka. It was initially conducted in the Ragama Medical Officer of Health area of the Gampaha district in 2007. The study population was adults aged 35–64 years, chosen by age-stratified random sampling from electoral lists. This cohort was re-evaluated in 2014 as part of a 7-year follow up study. We previously reported a prevalence of 38.9% for MetS from the 2007 RHS initiation cohort [10]. The aim of the present study was to assess the incidence and risk factors of new-onset MetS in the same cohort after 7 years of follow-up.

## Methods

This study was part of an on-going, large, community-based cohort follow up study: the Ragama Health Study (RHS) [9]. Ethical approval for the study was obtained from the Ethical Review Committee of the Faculty of Medicine, University of Kelaniya, Ragama, Sri Lanka. (Approval No—P/38/09/2006 and P/169/08/2014). The study was conducted in the Ragama Medical Officer of Health (MOH) administrative area situated 18 km north of the commercial capital, Colombo. Ragama has urban characteristics and a multi-ethnic population. The study population consisted of resident adults, originally selected by age-stratified random sampling from electoral lists. The target population was screened initially in 2007 (aged 35–64 years) and subsequently invited back after 7 years (aged 42–71 years) for re-evaluation in 2014.

On both occasions all participants were assessed using a structured interview, clinical and anthropometric measurements, liver ultrasound, and biochemical and serological tests. Details of screening of the inception cohort are described elsewhere [9]. Trained medical personnel interviewed the participants at re-evaluation, obtaining information on socio-demographic variables and lifestyle habits, including diet, physical activity (PA) and alcohol consumption. Past medical records of the subjects were analysed and recorded. Height was measured using a wall-mounted stadiometer to the nearest 0.1 cm and weight was measured (in light indoor clothing) using a digital scale to the nearest 0.1 kg. Waist circumference (WC) was measured at the midpoint between the inferior border of the ribcage and the superior iliac crest, and hip circumference was measured at the level of the trochanters. Waist and hip circumferences were measured to the nearest 0.1 cm using an inelastic measuring tape. Blood pressure was measured from the right upper limb in the sitting position using an Omron 705CP automatic blood pressure monitor and mean value of two readings taken 5 min apart was recorded.

Change in WC was classified as reduction (> 5% of initial measurement), no change (reduction ≤ 5% to increase < 5%) and increase (≥ 5% of initial measurement). Change in weight was classified as loss (> 5%), no change (loss ≤ 5% to gain < 5%), gain ≥ 5% and gain ≥ 10%.

Total body fat (TBF) and visceral fat percentage (VFP) were measured, during re-evaluation in 2014, with a body composition monitor using a proven bioelectrical impedance method (Omron HBF-362 body composition monitor, Omron Healthcare, Lake Forrest, Illinois, United States). Abnormal TBF was defined for females as > 32% and for males as > 25%, while abnormal VFP for both females and males was defined as > 10%. MetS was diagnosed on International Diabetes Federation (IDF 2006) criteria [1].

A 10-mL sample of venous blood was obtained from each subject. This was used to determine glycosylated hemoglobinA1c (HbA1c), fasting serum triglycerides (TG) and high density lipo-proteins (HDL), serum alanine aminotransferase activity (ALT) and hepatitis B and C serology [hepatitis B surface antigen (HBsAg) and anti-hepatitis C virus antibodies (anti-HCV) using CTK Biotech ELISA kits]. All subjects underwent ultrasonography of the liver with a 5-MHz 50 mm convex probe (MindrayDP-10 Ultrasound Diagnostic Systems, Mindray Medical International Limited, Shenzhen, China). Five trained medical officers carried out liver ultrasonography. Non-alcoholic fatty liver disease (NAFLD) was diagnosed on established ultrasound criteria for fatty liver, in the absence of any secondary cause for fatty liver [9].

Data were entered in Epi Info 7 (Centres for Disease Control and Prevention, Atlanta, GA, USA) and logical and random checks were done. Statistical analysis was done using R programming language version 3.5.1. Continuous and categorical data were described using median and inter quantile range (IQR) and frequencies (percentages), respectively. Groups comparison were done using Wilcoxon Rank Sum test and Pearson’s Chi-square-test. Variables that are associated with new onset of MetS were identified in a two-step process; initially, individual variables were screened using Wilcoxon rank sum test and Pearson’s Chi-square-test and variables which showed association at P = 0.2 level were considered as the potential variables for model fitting. Subsequently, these variables were considered to develop a generalized linear model to identify the variables that were associated with new onset MetS. Model fit was assessed with residual plots and ratio between residual deviance and residual degrees of freedom. P < 0.05 was considered as significant.

## Results

2985/3012 (99.1%) [1636-women (54.8%); median age (IQR) 53 (47–59) years] of the original study participants had complete data for analysis. 2148/2985 (71.9%) of the original cohort attended follow-up [1237 (57.5%) women; median age (IQR) 60 (54–66) years]. Initial and follow-up cohorts were similar, except for fewer males and more participants having central obesity in the follow-up cohort (Table 1).

**Table 1:** Profile of the participants in 2007 who attended and did not attend follow up in 2014

	Attended follow-up in 2014	Did not attend follow-up 2014	P value
	n = 2148	n = 837	
Males (%)	910 (42.4)	439 (52.4)	< 0.001
Median age (IQR)	53.0 (47.0–59.0)	53.0 (46.0–59.7)	0.630
Central obesity	1213 (56.5%)	405 (48.4%)	< 0.001
Median FBS (IQR)	104.0 (96.0–117.0)	104.5 (97.0–124.0)	0.035
Median SBP (IQR)	132.0 (119.0–147.0)	132.0 (120.0–147.0)	0.461
Median DBP (IQR)	78.0 (71.0–87.0)	79.0 (72.0–87.9)	0.340
Median TG (IQR)	115.0 (85.0–162.0)	121.0 (86.0–161.0)	0.226
Median HDL (IQR)	50.0 (48.0–52.0)	50.0 (48.0–52.0)	0.759

*IQR* inter quantile range, *FBS* fasting blood sugar, *SBP* systolic blood pressure, *DBP* diastolic blood pressure, *TG* triglyceride, *HDL* high density lipoprotein

949 participants [701 females (73.9%); median age (IQR) 60 (54–65) years] had MetS in 2014. Overall prevalence of MetS in 2014 was 47.2% (95% CI 45.0–49.4%), with a prevalence of 28.6% (95% CI 25.6–1.6%) among males and 61.4% (95% CI 58.6–64.2%) among females.

**Fig. 1:**
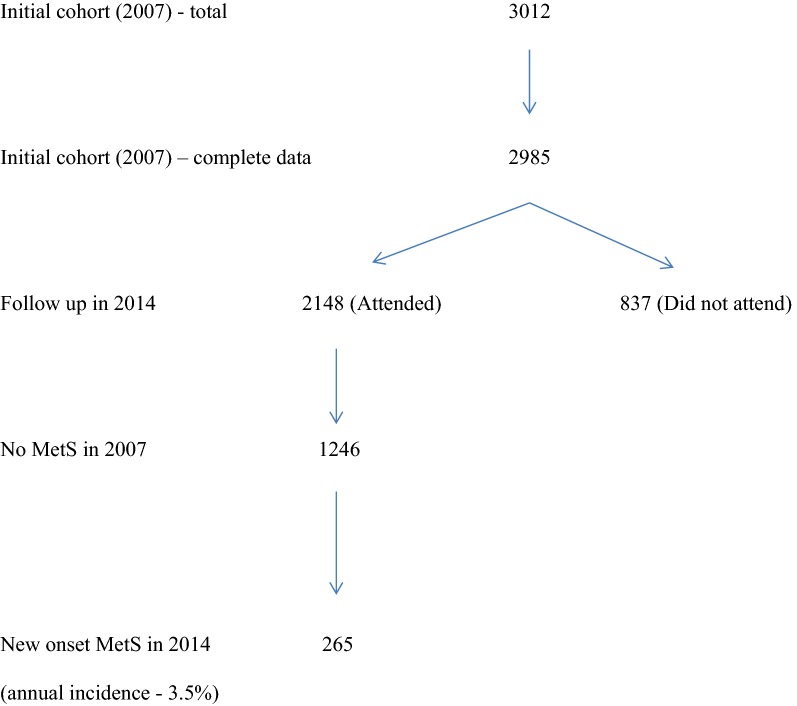
Study population

Out of 1246 participants who did not have MetS in 2007 and attended follow up in 2014, 265 [178 females (67.1%), median age (IQR) 57 (51–64) years] had developed new-onset MetS after 7 years, giving an annual incidence rate of 3.5% (95% CI 2.4–4.5%) (Fig. 1).

Table 2 shows a comparison between those with and without new-onset MetS in 2014, with regard to their characteristics at baseline (2007). Those who developed new-onset MetS were likely to be female, and have a higher BMI, central obesity and NAFLD in 2007. Any increase in weight and waist circumference from baseline was also significantly more likely to be associated with development of new-onset MetS.

**Table 2:** Characteristics of participants in 2007 and the presence and absence of new-onset MetS in 2014

Characteristic in 2007	New-onset MetS in 2014 n = 265	No MetS in 2014 n = 875	P value
Females (%)	178 (67.2%)	292 (35.1%)	< 0.001
Median age (IQR)	50.0 (44.0–57.0)	53.0 (47.0–58.0)	0.037
BMI > 23 kg/m^2^	174 (65.9%)	211 (25.4%)	< 0.001
Increase in weight (%)			
< 2	87 (33.1%)	485 (58.5%)	< 0.001
2–5	132 (50.2%)	220 (26.5%)	< 0.001
> 5	44 (16.7%)	124 (15.0%)	< 0.001
Central obesity	101 (38.2%)	87 (10.5%)	< 0.001
Increase in waist (%)			
< 2	59 (22.3%)	548 (66.0%)	< 0.001
2–5	52 (19.6%)	109 (13.1%)	< 0.001
> 5	154 (58.1%)	174 (20.9%)	< 0.001
Raised plasma glucose	206 (77.7%)	707 (84.6%)	0.013
Raised blood pressure	104 (39.4%)	350 (42.2%)	0.468
Raised triglycerides	58 (21.9%)	188 (22.6%)	0.861
Reduced HDL cholesterol	63 (24.4%)	138 (16.7%)	0.011
NAFLD in 2007	72 (27.2%)	100 (12.0%)	< 0.001

*IQR* inter quantile range, *BMI* body mass index, *HDL* high density lipoprotein, *NAFLD* non-alcoholic fatty liver disease

Parameter estimates of the fitted generalized model for new-onset MetS is given in Table 3. New-onset MetS was associated with female gender, BMI > 23 kg/m^2^ and NAFLD in 2007, weight gain and increase in waist circumference from 2007 to 2014. Females compared to males (OR = 4.9, 95% CI 3.4–7.4), those with BMI > 23 kg/m^2^ (OR = 1.6 per unit increase, 95% CI 1.5–1.7) and NAFLD (OR = 1.70, 95% CI 1.04–2.76) in 2007 had a higher risk of developing MetS.

**Table 3:** Parameter estimates of the fitted generalized linear model for incident MetS

Parameter	Estimate	Std. error	Z value	P value
Intercept	− 14.36	1.03	− 13.90	< 0.001
Females compared to males	1.60	0.20	7.99	< 0.001
BMI > 23 kg/m^2^ in 2007	0.46	0.04	11.57	< 0.001
Increase in weight				
2–5% compared to < 2%	0.69	0.29	2.39	0.017
> 5% compared to < 2%	0.77	0.23	3.36	< 0.001
> 5% compared to < 2–5%	0.09	0.28	0.31	0.756
Increase in waist circumference (reference level < 2%)				
2–5% compared to < 2%	1.94	0.28	6.83	< 0.001
> 5% compared to < 2%	2.61	0.26	10.22	< 0.001
> 5% compared to < 2–5%	0.67	0.25	2.65	0.008
NAFLD 2007	0.53	0.25	2.14	0.03

*BMI* body mass index, *NAFLD* non-alcoholic fatty liver disease

Compared to those with a weight gain of less than 2% from baseline in 2007, participants who gained 2–5% weight (OR = 2.0, 95% CI 1.1–3.5) and more than 5% weight (OR = 2.2, 95% CI 1.4–3.4) had higher risk of developing MetS. However, there was no difference in the risk between those who gained 2–5% and more than 5% weight (OR = 1.1, 95% CI 0.6–1.9). Compared to those who had waist circumference increase less than 2% from baseline in 2007, participants who had an increase of 2–5% (OR = 7.0, 95% CI 4.0–12.2) and more than 5% (OR = 13.4, 95% CI 8.3–22.4) had a higher risk of developing MetS. Risk of MetS was higher among those who had more than 5% increase in waist circumference since 2007, compared to those who had an increase of 2–5% (OR = 2.0, 95% CI 1.2–3.2).

**Fig. 2:**
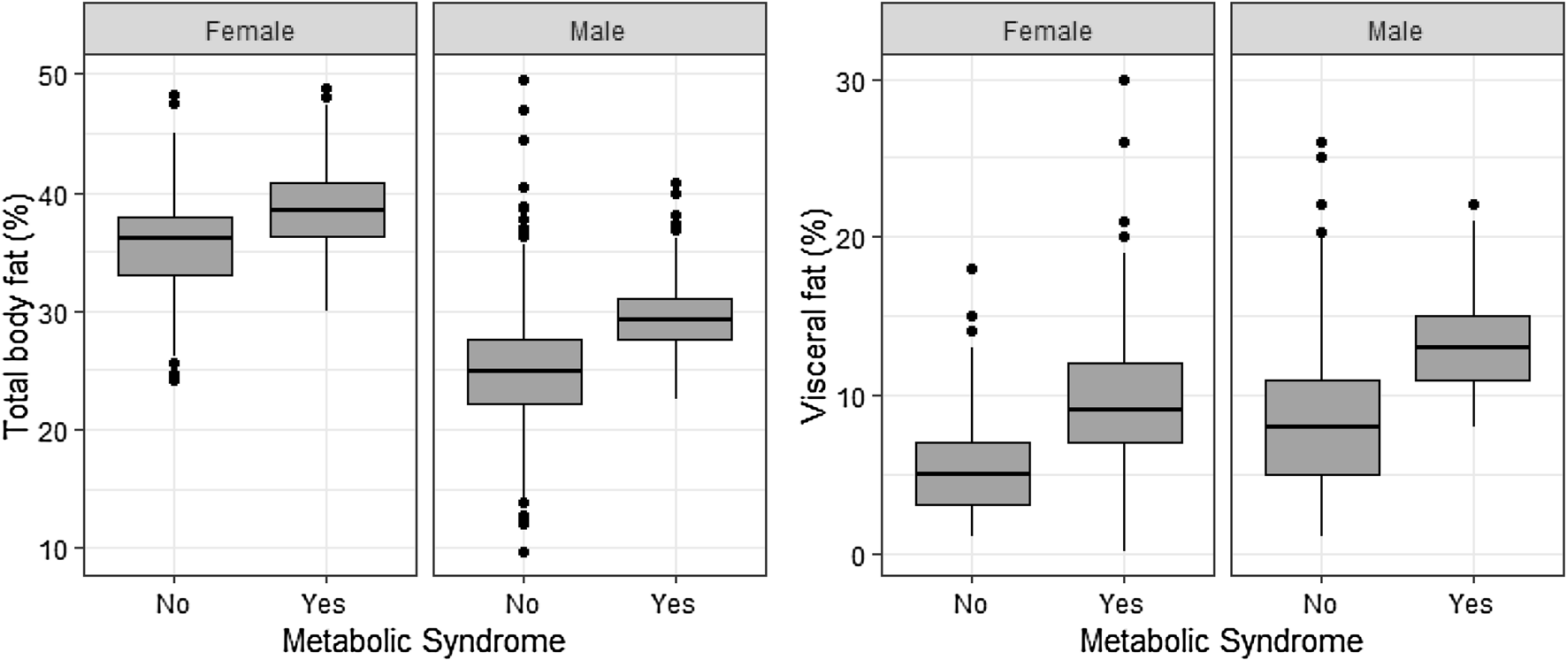
Distribution of total body fat percentage and visceral fat percentage among males and females with respect to MetS status

Both males and females with MetS had a higher median (IQR) total body fat percentage in 2014 compared to those without [females: 38.5% (36.3–40.9%) vs. 36.2% (33.0–37.9%), P < 0.001; males: 29.2% (27.6–31.2%) vs. 24.9% (22.2–27.7%), P < 0.001]. Those with new-onset MetS had a higher percentage of total body fat (77.9% vs. 60.0%, P < 0.001), and a higher visceral fat content (51.9% vs. 18.4%, P < 0.001), compared to those without new-onset MetS (Fig. 2).

## Discussion

In this community cohort follow-up study, the annual incidence of MetS was 3.5%. Female gender, BMI > 23 kg/m^2^ and NAFLD in 2007 and increase in weight and waist circumference from baseline were predictive of new-onset MetS. Abnormal TBF and VPF in 2014 were also significantly more likely in those with new-onset MetS.

In 2007 we reported a community prevalence of 38.9% for MetS in this urban, adult Sri Lankan population [10]. By 2014, 28.6% of men and 61.4% of women had MetS, giving a combined prevalence of 47.2%, which is almost half the study population. This is a higher community prevalence of MetS than previously reported from the region: Asia–Pacific 11.9–37.1% [4], South-Asia 26.1% [5] and Sri Lanka 26.1–34.8% [6], and may be explained by the increasing mean age of the study population by 2014 with a cumulative effect for development of MetS. The prevalence of MetS appears to vary according to the degree of urbanization, lifestyle and other socio-cultural parameters, such as income and level of education. Recent data indicate that approximately a third of the urban population in many large cities in South Asia have MetS [11, 12]. The MetS prevalence rates of other regions from previous reports are also comparatively lower than what is reported here: Africa 12.5–62.5% [13], Central America 23.0–35.1% [14], Europe 11.6–26.3% [15], Middle East 13.6–36.3% [16], South America 18.8–43.3% [17], and worldwide 20–25% [3].

There are previously reported studies on incidence of MetS from some parts of Asia. Hwang et al. [8] followed up a cohort of 1095 subjects in semi-urban Korea for 5 years and reported a cumulative MetS incidence of 3.5%, which is similar to the incidence reported in our study. Hwang et al. also noted that the presence of components of MetS at baseline were predictive of future development of MetS, a feature also seen in our study. In a study of 2858 Iranian adults Hadaegh et al. [7] reported a MetS incidence of 5.5%, over 9.3 years of follow up. In this study too predictors of developing incident MetS included individual components of MetS. Ours is the first to report the incidence and risk factors for MetS from a community based, cohort follow-up study in a South Asian population. Our reported incidence of 3.5% is comparable to previous reports in selected Asian populations.

In a more recent study from Europe, Van Ancum et al. reported about predictors of MetS in community-dwelling adults aged 55–85 years in the Netherlands over a 3-year period [18]. A higher BMI was predictive of developing MetS in this longitudinal cohort of 218 participants, although incidence was high at 30%.

In the inception population in 2007, we reported that the presence of MetS was associated with older age, female sex, worsening glycaemic control and obesity [10]. At re-assessment 7 years later, female sex, BMI > 23 kg/m^2^, NAFLD and increase in weight and waist circumference strongly predicted the development of new onset MetS. In addition, increased TBF and VPF were also associated with new-onset MetS. Therefore, obesity, whether central or general, appears to be the pivotal risk factor for the development of MetS.

Our study showed an association between the presence of NAFLD in 2007 and the development of MetS in 2014. Previous studies have also shown an association between NAFLD and the presence of MetS, and NAFLD has been proposed as a novel criterion for the diagnosis of MetS [12]. NAFLD may be the hepatic manifestation of MetS and insulin resistance is likely to be the main pathogenetic mechanism. Hyperinsulinemia is probably the consequence rather than cause of NAFLD and NAFLD can be considered an independent predictor of cardiovascular disease [19].

The strengths of our study are in its design as a true community-based, prospective, cohort follow-up with a reasonable number attending follow-up after 7 years. Furthermore, almost all demographic characteristics were similar in the inception and follow-up cohorts. There were, however, several limitations in our study. One was that information on TBF and VPF were not available at baseline for risk assessment. Another was that our follow-up sample consisted of mainly older adults. However, it is well-known that prevalence of MetS increases with older age and the dominant components may also differ from those seen in younger populations [20]. Although the diagnosis of NAFLD was based on accepted criteria (rather than surrogate markers such as hepatic enzymes), inter-observer variability of the operators was not formally assessed. Due to the unreliability of the collected data related to dietary habits and physical activity, we could not investigate the effects of diet and exercise on the development of MetS in this population. This is a major limitation of this study, which we hope to address during the next follow-up of the study cohort.

## Conclusion

This study demonstrates that the incidence and prevalence of MetS in urban Sri Lankan adults are high and is comparable to those reported in other parts of Asia [21]. The strong association of incident MetS with obesity and its markers suggests that preventive strategies should be aimed at control of obesity in the community. Early identification obesity coupled with emphasis on healthy lifestyle, diet and regular physical activity at community level is the best approach to prevent the development of MetS.

## Data Availability

The datasets used and analyzed during the current study are available from the corresponding author on reasonable request.

## Abbreviations

ALT: serum alanine aminotransferase
anti-HCV: anti-hepatitis C virus antibodies
BMI: body mass index
BP: blood pressure
CI: confidence interval
CVD: cardiovascular disease
DBP: diastolic blood pressure
FBS: fasting blood sugar
HbA1c: glycosylated hemoglobinA1
HBsAg: hepatitis B surface antigen
HDL: high density lipoprotein
IDF: International Diabetes Federation
IQR: inter quantile range
MetS: metabolic syndrome
MOH: Medical Officer of Health
NAFLD: non-alcoholic fatty liver disease
OR: odds ratio
PA: physical activity
RHS: Ragama Health Study
SBP: systolic blood pressure
TBF: total body fat
TG: triglyceride
VFP: visceral fat percentage
WC: waist circumference
